# Neutrophil-to-Lymphocyte Ratio and Albumin: New Serum Biomarkers to Predict the Prognosis of Male Alcoholic Cirrhosis Patients

**DOI:** 10.1155/2020/7268459

**Published:** 2020-12-21

**Authors:** Mingyuan Zhang, Yu Zhang, Lili Liu, Mozumder Prithweeraj, Hongqin Xu, Ruihong Wu, Xiaoyu Wen, Junqi Niu

**Affiliations:** Department of Hepatology, First Hospital of Jilin University, Xinmin Street, No. 71, Changchun, Jilin Province 130021, China

## Abstract

**Background and Aims:**

Alcohol-associated liver disease is exhibiting an increasing disease burden. In terms of pathogenesis, inflammation is closely related to alcohol-induced liver injury. The neutrophil-to-lymphocyte ratio (NLR) is a novel inflammatory biomarker. Here, we aim to evaluate the role of the NLR and other biomarkers in predicting short-term mortality in alcoholic cirrhotic patients.

**Methods:**

This was a retrospective study that included 459 male alcoholic cirrhosis patients. Among them, 345 patients completed follow-up. Based on their 30-day mortality information, patients were separated into surviving and nonsurviving groups. Demographic, clinical, and biochemical features were collected for further analysis. Logistic regression was used to identify factors associated with short-term mortality, and receiver operating characteristic (ROC) curve analysis was used to establish the predictive value of these factors.

**Results:**

The prognostic scores were significantly higher in the nonsurviving group than in the surviving group: NLR: 5.5 vs. 3.2 (*P* < 0.001), model for end-stage liver disease (MELD): 15.4 vs. 7.9 (*P* < 0.001), Maddrey's discriminant function (MDF): 39.8 vs. 12.7 (*P* < 0.001), and the integrated MELD (i-MELD): 37.9 vs. 28.4 (*P* < 0.001). Logistic regression demonstrated that albumin (ALB), NLR, and i-MELD values were significantly correlated with patient death in 30 days. On ROC analysis, the diagnostic accuracy for 30-day mortality of the NLR (area under the ROC curve (AUROC) of 0.72, *P* < 0.001) was similar to that of the MELD or i-MELD (AUROCs of 0.71 and 0.74, respectively, *P* < 0.001). The new biomarker, NLA, calculated as 100 × NLR/ALB, had the best prognostic value. The cutoff values of the NLR and NLA for predicting 30-day mortality were 4.2 and 19.6, respectively.

**Conclusions:**

The NLR and its related biomarker NLA are simple and robust predictors of 30-day mortality in alcoholic cirrhosis patients.

## 1. Introduction

Alcoholic liver disease (ALD) includes a complex spectrum of conditions ranging from steatosis to cirrhosis and imposes a substantial burden on public health [1]. Globally, disability-adjusted life years (DALYs) attributable to alcohol increased by more than 25% between 1990 and 2016, driven primarily by increased alcohol consumption in South Asia, Southeast Asia, and Central Asia [2]. Cirrhosis is the leading cause of liver-related deaths in the Asia-Pacific region, and deaths due to cirrhosis in this region represented 54.3% of cirrhosis-related deaths globally in 2015 [3]. As reported, in 2015, alcohol consumption accounted for 20.0% of all deaths due to cirrhosis and other chronic liver diseases and for 35.5% of all deaths due to liver cancer in mainland China [4]. Most patients with ALD are diagnosed at the decompensation stage [5].

Prognostic biomarkers of ALD have been the focus of many research studies [6]. Several scoring systems for risk stratification are currently used in practice. Child-Pugh and model for end-stage liver disease (MELD) scores have been widely explored as prognostic models for outcomes of chronic liver cirrhosis [7, 8]. In addition, Maddrey's discriminant function (MDF) is commonly used to predict 28-day mortality and identify severe alcoholic hepatitis. The integrated-MELD (i-MELD), which incorporates serum sodium and age, could better predict 12-month mortality than the original MELD, but the calculation process is more complicated [9–11].

Recently, researchers have attempted to discover new prognostic models by not only studying easily accessible specimens such as circulating blood markers but also calculating such models, including the gamma-glutamyl transpeptidase-to-platelet ratio (GPR), which could predict 90-day mortality in patients with acute-on-chronic liver failure [12], and the red blood cell distribution width (RDW), which was reported to be independently associated with poor hospital outcome [11]. Among these models, the neutrophil-to-lymphocyte ratio (NLR) might be an eligible candidate biomarker and might be comparable to Child-Pugh and MELD scores in predicting outcomes in cirrhotic patients [13–16]. However, few studies have assessed whether such simple biomarkers can predict the outcome of alcoholic patients with cirrhosis.

In the present study, we retrospectively analyzed the medical records of alcoholic cirrhosis patients in the First Hospital of Jilin University from September 2004 to December 2019. We aimed to evaluate and identify simple and reliable prognostic models for predicting the 30-day mortality of alcoholic cirrhosis patients.

## 2. Materials and Methods

### 2.1. Patients and Study Design

**Figure 1:**
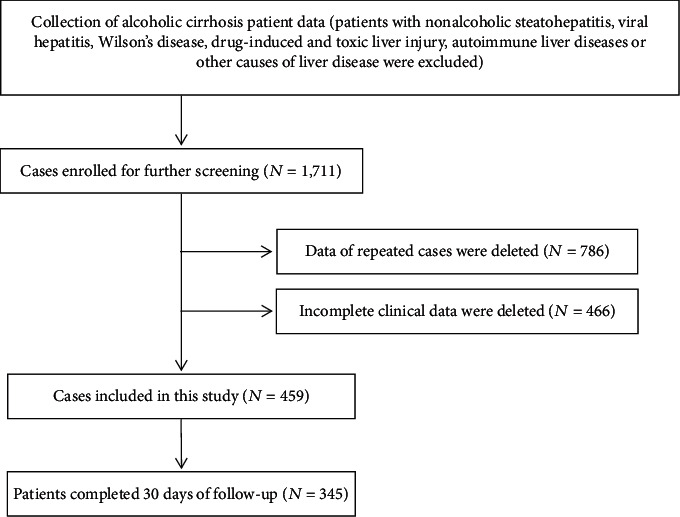
Study screening flowchart.

This was a single-center retrospective study; records of patients diagnosed with alcoholic cirrhosis were collected. Alcohol consumption histories were based on the patients' and relatives' reports, which were recorded by their physicians. Patient symptoms and signs were obtained from their medical files. Diagnostic criteria for alcoholic cirrhosis were as previously described [17]. In this period, as there were few female patients who developed alcoholic cirrhosis in our center, we enrolled only adult male patients. In total, 459 patients with complete demographic and laboratory parameter data were collected. According to clinical and biochemical parameters present on admission and noninvasive scores, including the MDF, MELD, i-MELD, NLR, and GPR, were calculated, and RDW levels on admission were recorded. By checking the medical records and telephone follow-up, we collected the 30-day mortality data of patients in this cohort. All laboratory analyses were performed at the First Hospital of Jilin University, data were collected from the electronic medical record system, and patients were anonymous (Figure 1). The study was approved by the Ethics Committee of First Hospital of Jilin University and performed in accordance with the Declaration of Helsinki.

Continuous variables are expressed as medians (interquartile range [IQR]) and were compared by a Mann–Whitney test), while categorical variables are presented as numbers (percentages) and were compared by the Pearson Chi-square test. Logistic regression analysis was applied to identify factors associated with a high risk of short-term mortality, and odds ratios (ORs) with 95% confidence intervals (CIs) were used to determine the strength of statistical associations. Receiver operating characteristic (ROC) curve analysis was used to establish the value of noninvasive markers in predicting the occurrence of 30-day mortality. The validity of the cutoff point was measured via the area under the ROC curve (AUC). SPSS Statistics 23 software (IBM ® Corporation, USA) was used for statistical analyses. For all analyses, a two-sided *P* value less than or equal to 0.05 was considered significant.

## 3. Results

### 3.1. Patient Demographics and Clinical Features

The baseline demographic profiles and clinical characteristics of the study population were recorded. The 30-day mortality data of patients were collected by medical records and telephone follow-up; however, because this was a retrospective study, it was difficult to contact some patients after they were discharged from our center. Ultimately, 114 patients did not have follow-up data; therefore, we obtained 30-day mortality information from 345 patients. Among them, 40 patients who died within 30 days were assigned to the nonsurviving group, and the other 305 patients who lived more than 30 days were assigned to the surviving group. To avoid bias that might be induced by missing records, we compared the patient demographic information, laboratory parameters, and prognostic scores between patients who were followed up and those lost to follow-up. The results demonstrated that all parameters were similar between the two groups (Table 1); therefore, we assumed that the patients who were followed up represented the whole cohort.

Data are provided as the median (IQR) and number of cases (%); AST: aspartate aminotransferase; ALT: alanine aminotransferase; ALP: alkaline phosphatase; GGT: gamma-glutamyl transpeptidase; TBIL: total bilirubin; ALB: albumin; RDW: red blood cell distribution width; GPR: gamma-glutamyl transpeptidase-to-platelet ratio; NLR: neutrophil-to-lymphocyte ratio; MELD: model for end-stage liver disease; i-MELD: integrated-MELD; MDF: Maddrey's discriminant function; normal ranges: AST 8-40 U/L; AST 8-50 U/L; ALP: 15-112 U/L; GGT: 5-54 U/L; TBIL: 6.8-30.0 *μ*mol/L; DBIL: 0.0-8.6 *μ*mol/L; AChE: 4300-12 000 U/L; ALB: 35-55 g/L; PLT: (100 − 300) × 10^9^/L; RDW: 11.0%-14.0%.

Next, we compared the demographic profiles, definite clinical features, and laboratory results of patients in the surviving and nonsurviving groups, and the results indicated no significant differences in age, daily intake of alcohol, or lifetime duration of drinking between the two groups. On admission, clinical features such as fatigue and abdominal distension were similar between groups. Compared with those in the surviving group, patients in the nonsurviving group had a higher incidence of hepatic encephalopathy (45.0% vs.13.8%, *P* < 0.001), and there were more Child-Pugh C stage patients in this group (85.0% vs.55.1%, *P* < 0.001) (Table 2).

Data are provided as the median (IQR) and number of cases (%); HCC: hepatocellular carcinoma; AST: aspartate aminotransferase; ALT: alanine aminotransferase; ALP: alkaline phosphatase; GGT: gamma-glutamyl transpeptidase; TBIL: total bilirubin; ALB: albumin; PLT: platelets; PT: prothrombin time; normal ranges: AST 8-40 U/L; AST 8-50 U/L; ALP: 15-112 U/L; GGT: 5-54 U/L; TBIL: 6.8-30.0 *μ*mol/L; DBIL: 0.0-8.6 *μ*mol/L; AChE : 4300-12 000 U/L; ALB: 35-55 g/L; PLT: (100 − 300) × 10^9^/L; RDW: 11.0%-14.0%.

### 3.2. Laboratory Features

Differences in laboratory parameters between the surviving and nonsurviving groups are demonstrated in Table 2. Furthermore, we calculated the prognostic scores that were previously mentioned and compared the surviving and nonsurviving groups (Table 3).

*Calculation formulas*:

MELD = 3.8 × log (bilirubin mg/dl) + 11.2 × log (INR) + 9.6 × log (creatinine mg/dl) + 6.4 [7]

i − MELD = MELD + (age × 0.3) − (serum sodium × 0.7) + 100 [11]

MDF = 4.6 × (PT − control PT) + bilirubin (mg/dl) [9]

NLR = neutrophils/lymphocytes

GPR = (GGT/upper limits of normal)/PLT, to facilitate the calculation and description, the value was multiplied by 100

Data are provided as the median (IQR); RDW: red blood cell distribution width; GPR: gamma-glutamyl transpeptidase-to-platelet ratio; NLR: neutrophil-to-lymphocyte ratio; MELD: model for end-stage liver disease; i-MELD: integrated-MELD; MDF: Maddrey's discriminant function.

A logistic regression model was used to identify factors that might be associated with high risk for short-term mortality. As total bilirubin and prothrombin time were used to calculate MDF and the MELD was used to calculate the i-MELD, to avoid the effect of collinearity, these indicators were not included in the model. We finally included albumin (ALB), MDF, NLR, and i-MELD in the logistic regression model, and the ORs and 95% CIs were calculated. The logistic regression results demonstrated that ALB, NLR, and i-MELD were significantly associated with death. The ORs of ALB, NLR, and i-MELD are shown in Table 4.

Data are provided as the median (IQR); ALB: albumin; NLR: neutrophil-to-lymphocyte ratio; i-MELD: integrated-MELD.

### 3.3. Prognostic Markers for 30-Day Mortality in ALD Patients

ROC curve analysis was used to evaluate the sensitivity and specificity of ALB and NLR for predicting 30-day mortality in ALD patients. We also evaluated the predictive abilities of the MELD, i-MELD, and MDF scores for comparison. The results demonstrated that the ALB level was negatively correlated with mortality. Using this parameter, we designed a new model called NLA, which is calculated as 100 × NLR/ALB, and the reanalysis results showed that NLA had the best prognostic value among these prognostic scores (Table 5).

**Figure 2:**
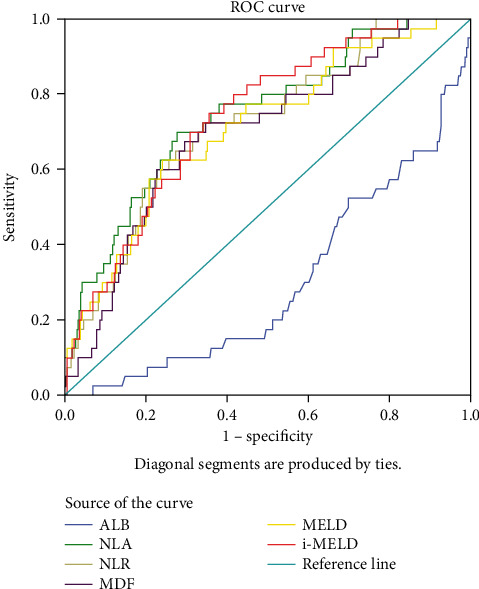
ROC curves of prognostic markers for predicting 30-day mortality.

Cutoff points were determined by the maximum sum of sensitivity and specificity. The cutoff value of the NLA was 19.6 (yielding sensitivity and specificity values of 70.0% and 72.5%). These results showed that the NLR and its derived parameter NLA might be regarded as simple and superior prognostic biomarkers for cirrhosis patients with ALD (Figure 2).

Data are provided as the median (IQR); MELD: model for end-stage liver disease; i-MELD: integrated-MELD; MDF: Maddrey's discriminant function; NLR: neutrophil-to-lymphocyte ratio; NLA:100 × (neutrophil − to − lymphocyte ratio)/albumin.

## 4. Discussion

In this retrospective study, we show that the NLR and blood ALB level are significantly correlated with disease progression and survival in alcoholic cirrhosis patients. The indicator NLA, which is calculated by combining the NLR with ALB, can better predict patients' 30-day mortality than the MELD, MDF, and i-MELD scores. Indeed, an NLA > 19.6 predicted that alcoholic cirrhosis patients would have a high risk of mortality within 30 days.

Some scoring models have been proposed to predict the prognosis and mortality of liver cirrhosis patients, including the Child-Turcotte-Pugh (CTP) and MELD scores, which are well established and the most widely used models. Other scoring systems include the i-MELD, ALB, bilirubin, international normalized ratio (INR) and creatinine score (ABIC), and RDW. The Child-Pugh score is calculated according to serum levels of bilirubin and ALB, prothrombin time, degree of ascites, and severity of hepatic encephalopathy [18]. The MELD score is calculated by combining the serum levels of creatinine and bilirubin and the INR. However, there are drawbacks for the calculation of Child-Pugh and MELD scores, as the Child-Pugh score requires subjective indexes, such as hepatic encephalopathy and ascites. For retrospective studies, it is difficult to evaluate these factors, and the results are easily influenced by the use of lactose and diuretics. Regarding the MELD score or the modified i-MELD score, the calculation formulas are very complicated, thus limiting their wide application in practice [16].

The NLR is the ratio of neutrophils to lymphocytes, and the two indexes of the NLR can be easily obtained from simple blood tests in clinical practice. The NLR is a noninvasive biomarker that reflects systemic inflammation and the general nutrition status of patients, which has been shown to predict survival and prognosis in various patient populations with cardiac and malignant diseases. As reported, a preoperative elevated NLR significantly increased the risk of recurrence in patients who underwent liver transplantation for hepatocellular carcinoma (HCC), which may be related to the inflammatory tumor microenvironment [19–21].

The morbidity and progression of liver fibrosis and liver cirrhosis have been considered to be highly correlated with inflammation. In cirrhotic patients, immune and inflammatory systems are activated, and inflammatory markers, such as interleukin-6 and tumor necrosis factor-alpha, have been found to be elevated [22]. However, in liver cirrhosis patients, neutropenia and lymphocytopenia are associated with hypersplenism, but lymphocytopenia is more prominent because of malnutrition. Together, neutrophils and lymphocytes, as the NLR, may be a useful indicator for assessing the prognosis of patients with chronic liver diseases [23]. Biyik et al. performed a retrospective observational cohort study that included 145 cirrhotic patients, most of whom had viral hepatitis-related or cryptogenic cirrhosis. They found that the NLR could predict mortality in patients with liver cirrhosis independent of the CTP and MELD scores. In particular, the NLR could predict mortality in patients with low MELD and/or CTP scores [24].

In our research, we analyzed the prognostic value of the NLR in a large cohort of alcoholic cirrhosis patients, and the results showed that the NLR results were similar to those of the MELD and i-MELD, which was consistent with previous studies of cirrhosis patients. Furthermore, we identified the prognostic value of ALB in predicting patient short-term mortality in this cohort, combining the NLR with ALB. We invented a new index, the NLA, which had the best prognostic value. This new index is the main strength of our research, but the results still need to be further verified in a large external validation cohort.

This study has some limitations. First, as a retrospective study, a certain percentage of subjects were lost follow-up; however, to strengthen the data, we compared the clinical information of patients lost to follow-up with traceable patient information, and we found no difference between the two groups (Table 1). Second, the mortality information of patients was obtained by telephone follow-up and excluded suicide and traumatic accidents, although whether these mortalities were caused by other diseases could not be completely excluded. However, during hospitalization, ALD was the most severe condition; therefore, we suspected that most of these patients died from ALD. Third, the data presented here were from our single center, and the representativeness of this data might be limited; thus, other independent cohorts for further validation of NLR/NLA are still necessary. Forth, alcohol cessation is an important determinant of survival [3], and while most decompensated patients in this retrospective study may have stopped drinking, for patients with mild symptoms or compensated patients, it is difficult to obtain information on whether the patient had stopped drinking. Currently, it is difficult to formulate alcohol policies to manage alcoholic patients, and these measures still need to be improved.

## 5. Conclusion

In this study, we found that the NLR and a new biomarker, NLA, have robust prognostic value to predict 30-day mortality in alcoholic cirrhosis patients. However, further prospective studies are required to better elucidate the relationship between the NLR and NLA and patient mortality due to liver disease.

## Figures and Tables

**Table 1:** Baseline demographics and laboratory data of patients who were followed up or lost to follow-up.

Variables	Followed up (*N* = 345)	Lost to follow-up (*N* = 114)	*P* value
Basic demographics			
Age	51 (45-59)	49 (43-57.5)	0.10
Alcohol intake (g/day)	175 (125-250)	160 (125-250)	1.00
Duration of drinking (years)	27 (20-30)	25 (20-30)	1.00
Laboratory data			
AST (U/L)	60.7 (36.0-105.0)	67.2 (45.5-130.0)	0.06
ALT (U/L)	30.8 (21.2-47.8)	35.0 (24.5-51.0)	0.16
ALP (U/L)	112.5 (84.2-154.7)	108.0 (85.0-157.0)	0.89
GGT (U/L)	97.1 (43.2-303.8)	116.0 (56.0-349.7)	0.06
ALB (g/L)	26.1 (22.8-30.9)	25.2 (21.4-29.7)	0.15
TBIL (*μ*mol/L)	66.8 (31.8-160.8)	95.3 (35.2-205.6)	0.10
RDW	16.2 (14.8-18.3)	16.3 (14.7-17.6)	0.52
GPR	1.9 (1.0-4.4)	2.4 (1.0-6.6)	0.49
NLR	3.4 (2.0-5.4)	3.8 (2.1-6.9)	0.31
MDF	14.2 (2.4-35.4)	19.5 (4.61-46.6)	0.57
MELD	8.5 (2.8-14.5)	9.0 (2.9-15.0)	0.86
i-MELD	29.5 (22.9-37.3)	30.3 (22.3-37.6)	0.89
Child-Pugh A	24 (7.0%)	6 (5.3%)	0.66
Child-Pugh B	119 (34.5%)	36 (31.6%)	0.65
Child-Pugh C	202 (58.5%)	72 (63.1%)	0.44

**Table 2:** Baseline demographics and clinical characteristics of patients in the surviving and nonsurviving groups.

Variables	Surviving (*N* = 305)	Nonsurviving (*N* = 40)	*P* value
Basic demographics			
Age	51 (45-58)	50 (43-59)	0.25
Alcohol intake (g/day)	160 (125-250)	200 (125-250)	0.89
Duration of drinking (years)	27 (20-30)	25 (20-30)	0.99
Clinical features			
Anorexia and epigastric discomfort	59 (19.3)	7 (17.5)	1.00
Fatigue	134 (43.9)	18 (45.0)	1.00
Abdominal distension	172 (56.4)	29 (72.5)	0.06
Jaundice	122 (40.0)	18 (45.0)	0.61
Edema	108 (35.4)	13 (32.5)	0.86
Child-Pugh A	23 (7.5%)	1 (2.5%)	0.34
Child-Pugh B	114 (37.4%)	5 (12.5%)	0.001
Child-Pugh C	168 (55.1%)	34 (85.0%)	<0.001^∗∗^
Complications			
Hepatic encephalopathy	42 (13.8)	18(45.0)	<0.001^∗∗^
Ascites	232 (76.1)	31(77.5)	1.00
Gastrointestinal hemorrhage	61 (20.0)	10 (25.0)	0.53
HCC	8 (2.6)	1 (2.5)	1.00
Laboratory data			
AST (U/L)	60.3 (36.0-104.0)	66.0 (35.0-117.6)	0.43
ALT (U/L)	30.8 (22.0-47.0)	30.0 (16.0-52.0)	0.44
ALP (U/L)	114.0 (83.9-156.0)	108.9 (86.0-150.0)	0.46
GGT (U/L)	100.8 (45.0-317.6)	64.0 (31.0-186.0)	0.14
ALB (g/L)	26.9 (23.3-31.3)	24.1 (18.8-25.8)	0.001^∗∗^
TBIL(*μ*mol/L)	61.6 (31.0-131.0)	173.3 (72.1-315.8)	<0.001^∗∗^
PLT (×10^9^/L)	87.0 (55.0-140.0)	79.0 (55.0-125.0)	0.63
PT (s)	15.0 (13.4-18.1)	20.2 (15.0-22.8)	<0.001^∗∗^

**Table 3:** Comparisons of prognostic scores in the surviving and nonsurviving groups.

Prognostic score	Surviving (*N* = 305)	Nonsurviving (*N* = 40)	*P* value
RDW	16.2 (14.7-18.5)	16.4 (14.9-18.6)	0.68
GPR	2.0 (1.0-5.6)	1.7 (0.8-5.1)	0.38
NLR	3.2 (1.9-5.0)	5.5 (2.9-9.7)	<0.001^∗∗^
MELD	7.9 (2.25-13.2)	15.4 (8.9-21.2)	<0.001^∗∗^
MDF	12.7 (2.1-31.6)	39.8 (10.9-55.5)	<0.001^∗∗^
i-MELD	28.4 (22.2-35.9)	37.9 (30.9-47.2)	<0.001^∗∗^

**Table 4:** Multivariate regression of markers used to predict 30-day mortality.

Prognostic score	Odds ratio	95% confidence interval		*P* value
		Lower	Upper	
ALB	1.12	1.05	1.20	0.001
NLR	0.93	0.88	0.98	0.006
i-MELD	0/97	0.94	0.99	0.003

**Table 5:** Diagnostic value of prognostic scores in predicting 30-day mortality.

Prognostic score	Cutoff	Specificity	Sensitivity	PPV	NPV	AUC (95% CI)	*P* value
MELD	13.6	62.5%	76.1%	0.29	0.55	0.71(0.63-0.80)	<0.001^∗∗∗^
i-MELD	32.0	75.0%	64.3%	0.22	0.60	0.74(0.67-0.82)	<0.001^∗∗∗^
MDF	26.5	67.5%	70.5%	0.25	0.57	0.70(0.61-0.78)	<0.001^∗∗∗^
NLR	4.20	72.5%	65.6%	0.23	0.59	0.72(0.64-0.80)	<0.001^∗∗∗^
NLA	19.6	70.0%	72.5%	0.26	0.57	0.75(0.67-0.83)	<0.001^∗∗∗^

## Data Availability

This is a retrospective study. The data of this study are available from the corresponding author upon request.
